# Foundation models in bioinformatics

**DOI:** 10.1093/nsr/nwaf028

**Published:** 2025-01-25

**Authors:** Fei Guo, Renchu Guan, Yaohang Li, Qi Liu, Xiaowo Wang, Can Yang, Jianxin Wang

**Affiliations:** Hunan Provincial Key Lab on Bioinformatics, School of Computer Science and Engineering, Central South University, Changsha 410083, China; Xiangjiang Laboratory, Changsha 410083, China; Key Laboratory for Symbol Computation and Knowledge Engineering of the Ministry of Education, College of Computer Science and Technology, Jilin University, Changchun 130012, China; Department of Computer Science, Old Dominion University, Norfolk 23529, USA; School of Life Sciences and Technology, Tongji University, Shanghai 200092, China; Department of Automation, Tsinghua University, Beijing 100084, China; Department of Mathematics, State Key Laboratory of Molecular Neuroscience, and Big Data Bio-Intelligence Lab, The Hong Kong University of Science and Technology, Hong Kong, China; Hunan Provincial Key Lab on Bioinformatics, School of Computer Science and Engineering, Central South University, Changsha 410083, China; Xiangjiang Laboratory, Changsha 410083, China

**Keywords:** foundation model, bioinformatics, genomics, transcriptomics, proteomics, drug discovery, single-cell analysis

## Abstract

With the adoption of foundation models (FMs), artificial intelligence (AI) has become increasingly significant in bioinformatics and has successfully addressed many historical challenges, such as pre-training frameworks, model evaluation and interpretability. FMs demonstrate notable proficiency in managing large-scale, unlabeled datasets, because experimental procedures are costly and labor intensive. In various downstream tasks, FMs have consistently achieved noteworthy results, demonstrating high levels of accuracy in representing biological entities. A new era in computational biology has been ushered in by the application of FMs, focusing on both general and specific biological issues. In this review, we introduce recent advancements in bioinformatics FMs employed in a variety of downstream tasks, including genomics, transcriptomics, proteomics, drug discovery and single-cell analysis. Our aim is to assist scientists in selecting appropriate FMs in bioinformatics, according to four model types: language FMs, vision FMs, graph FMs and multimodal FMs. In addition to understanding molecular landscapes, AI technology can establish the theoretical and practical foundation for continued innovation in molecular biology.

## Teaser

This review presents recent advancements in bioinformatics Foundation Models (FMs) that employed in a variety of downstream tasks, including genomics, transcriptomics, proteomics, drug discovery, and single cell analysis.

## INTRODUCTION

Foundation models (FMs) represent large-scale artificial intelligence (AI) systems that undergo extensive pre-training on vast datasets, thereby enabling their application in a diverse array of downstream tasks. FMs are built by training neural networks on labeled and unlabeled data, enabling them to discern fundamental patterns and generalize knowledge to novel tasks. Before the emergence of foundation models, most AI systems were constructed using more traditional methodologies, which relied heavily on explicit human engineering and predefined rules rather than learning directly from data. The emergence of large-scale pre-trained models (PTMs) has fundamentally transformed the landscape of artificial intelligence. The field is currently undergoing a paradigm shift, propelled by the development of models trained on extensive datasets that can be applied across a diverse array of downstream applications. Foundation models present significant opportunities and inherent risks, arising from their capabilities and underlying technical principles, as well as their applications and societal implications [1]. As computational power and data availability continue to expand, significant breakthroughs are being achieved in four key areas: the design of effective architectures, the utilization of rich contextual information, the enhancement of computational efficiency and the execution of interpretative analysis. The development of FMs underscores the pivotal role of PTMs within the spectrum of AI technology.

As with pre-training architectures, a lot of large-scale foundation models are categorized into four AI model types: language FMs, vision FMs, graph FMs and multimodal FMs.

- *Language FMs.* Word2Vec [2] is an early PTM for converting words into distributed representations; transformers [3] deal with sequential data, training large language models (LLMs) and recurrent neural networks (RNNs); BERT [4] and GPT [5] are transformer-based PTMs that differ from word-level PTMs.
- *Vision FMs.* AlexNet [6] is a convolutional neural network (CNN) that has significantly advanced computer vision (CV); ResNet [7] introduces shortcut connections with residual layers trained on ImageNet; the segment anything model [8] is a promptable segmentation method that segments everything everywhere.
- *Graph FMs.* Graph neural networks (GNNs) are information processing architectures for emerging and homogenizing tasks; MPNN [9] and GIN [10] employ global and local temporal message-passing mechanisms; Graphormer [11] represents structural relationships between nodes using spatial encoding; GraphRAG [12] is a structured, hierarchical framework for retrieval augmented generation (RAG).
- *Multimodal FMs.* ViT [13] outperforms conventional supervised CNN in preliminary studies; CLIP [14] constructs a transformer-based multimodal PTM that demonstrates promising results.

**Figure 1.**
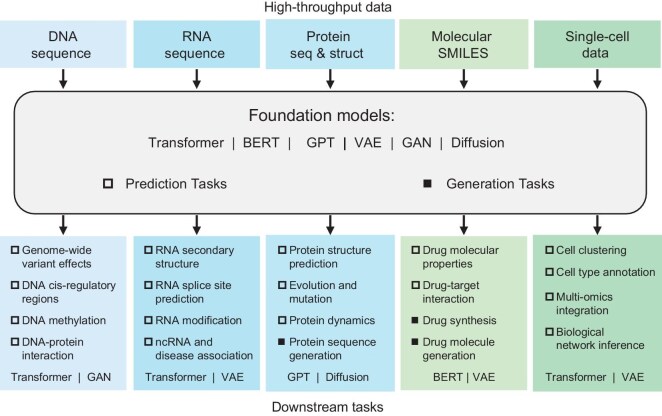
Foundation models in bioinformatics.

Recently, a number of foundation models have been successfully applied to bioinformatics problems, such as biomarker discovery, enzyme design, antibody-antigen recognition, drug discovery, omics analysis and disease diagnosis. The objective of this study is to provide an analysis of bioinformatics FMs that can be trained on both supervised and unsupervised learning models for applications such as core biological problems and integrated biological issues. With AI technology, it is possible to understand the molecular landscape, as well as aspects of human physiology and molecular biology. Several prominent foundation models are used to gain a deeper understanding of high-throughput biological data, followed by a discussion of how prediction and generation models have been applied at various downstream tasks in bioinformatics, as shown in Fig. 1.

Current surveys examine bioinformatics FMs from three perspectives. Several review articles summarize the large language models applied to bioinformatics tasks. Gao *et al.* [15] outlined transformer-based, bioinformatics-tailored foundation models, directly applied to biological sequence data and serializable data. Heider *et al.* [16] discussed large language models utilized to identify patterns in bioinformatics and analyzed their potential to revolutionize and accelerate multi-omics and personalized medicine discoveries. Moreover, a few survey papers enumerate the specific models for solving bioinformatics problems. Cheng *et al.* [17] summarized diffusion modeling frameworks used in computational biology to generate proteins, drugs and models of protein-ligand interactions. Furthermore, some review literature summarizes many traditional models in the field of bioinformatics and medicine. Li *et al.* [18] summed up current trends in deep learning models to examine specific biological challenges, evaluating their applications to sequence analysis, structure prediction and function annotation. Rajpurkar *et al.* [19] listed generalist medical AI models combining electronic health records, genomics, clinical texts and medical modalities. Despite this, most current surveys focus almost exclusively on one category of large-scale model or some traditional models that are applied to bioinformatics without taking into account various foundation models.

This review offers new insights into three primary objectives of foundation models in bioinformatics. First, we introduce recent improvements in bioinformatics foundation models as versatile tools. A comprehensive understanding of bioinformatics applications is provided by focusing on four types of foundation models: the language FM, vision FM, graph FM and multimodal FM. Second, we examine bioinformatics FMs for five downstream tasks, including genomics, transcriptomics, proteomics, drug discovery and single-cell analysis. Our discussion focuses on biological databases, training strategies, hyperparameter sizes and biological applications. Finally, we discuss our perspective on the promising trajectory of bioinformatics FMs, drawing on our experiences with model pre-training frameworks, benchmarking selections, whiteboxes and interpretability, and evaluating model hallucinations.

## EVOLUTION OF THE BIOINFORMATICS FM

**Figure 2.**
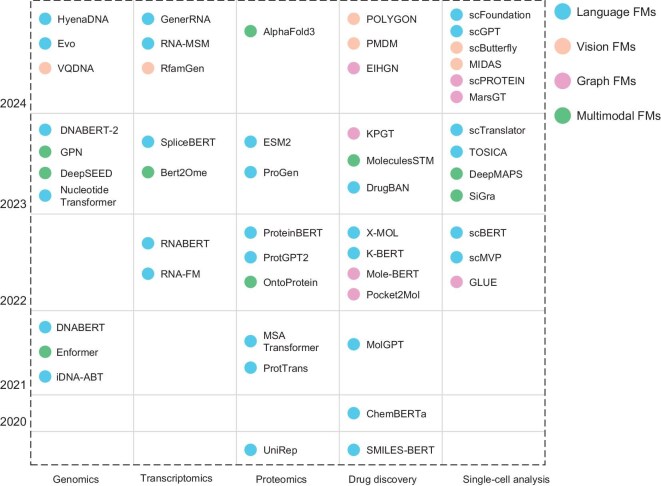
Evolution of FMs in bioinformatics.

The application of FMs in bioinformatics, which grew in popularity along with deep learning, was triggered by the introduction of large-scale pre-trained models. As a result of these efforts in bioinformatics, foundation models (language FMs, vision FMs, graph FMs and multimodal FMs) have shown promising results for applications in biology (genomics, transcriptomics, proteomics, drug discovery and single-cell analysis). The underlying bioinformatics foundation models need to be systematically reviewed, with a particular focus on various deep learning architectures. The evolution of FMs in bioinformatics is shown in Fig. 2.

The initial bioinformatics models primarily focused on specific prediction tasks, such as sequence classification (e.g. DNA/RNA/protein sequence annotation) and secondary structure prediction. These models were typically task specific and often developed from the ground up using domain-specific datasets. The subsequent phase in bioinformatics feature modeling involved the adoption of general pre-training strategies similar to those employed in natural language processing, such as BERT. Models like DNABERT [20] were created to leverage large-scale pre-training on genomic data, enabling them to capture broad, transferable features inherent in biological sequences. As feature models evolved, they began incorporating multi-task learning and transfer learning techniques, which allowed them to excel across a diverse array of biological tasks, including protein folding, genomic sequence analysis and drug discovery. The emergence of models like AlphaFold [21] for protein structure prediction exemplifies this era; these large-scale pre-trained models could be fine-tuned for highly specialized tasks, resulting in groundbreaking advancements within structural bioinformatics. The latest generation of bioinformatics foundation models is characterized by their multimodal capabilities, which facilitate the integration and reasoning over various types of biological data—including genomic, transcriptomic, proteomic and even clinical information. Models such as GLUE [22], originally developed for natural language processing tasks but subsequently adapted for bioinformatics applications like literature mining, and multitask deep learning frameworks designed for multi-omics data exemplify the growing trend towards multimodal learning.

By revealing the evolution process of the bioinformatics FM, it is possible to understand in depth how the revised model overcomes the limitations and shortcomings of the primary model. Taking advantage of the latest bioinformatics FM, one can achieve unprecedented accuracy, realize an integrated AI model and perform richer downstream analysis. Taking the classic biological problem ‘protein three-dimensional structure reconstruction’ as a representative demonstration, DeepMind has developed three iterations of an artificial intelligence system over the past five years. AlphaFold [21] was developed to predict protein structure models in 2020, whose prediction is far better than any other method. As a result of AlphaFold’s success, deep learning has enormous potential in the field of protein structure. In order to realize this process, AlphaFold follows two steps. First, AlphaFold predicts the distance and rotation distribution of residues by utilizing 220 deep residual convolution blocks. After that, the gradient descent on the potential-specific potential predicted in the first step is used to conclude the protein structure. Because of its two-step modeling, AlphaFold loses valuable information, especially in regard to the dependence between long-range residue pairs. Furthermore, AlphaFold2 [23] achieves high-precision predictions through two main components: ‘EvoFormer’ and ‘Structure module’. An innovative mechanism for exchanging information with the multiple sequence alignment (MSA) is used in the Evoformer block, which employs a number of novel attention-based components. A significant advancement in the field is the direct inference of paired representations encapsulating both spatial and evolutionary relationships. In contrast to AlphaFold1, the structure module uses invariant point attention to predict three-dimensional (3D) coordinates directly. Both AlphaFold1 and AlphaFold2 have attained outstanding performance in protein structure prediction, but their predictive prowess is augmented by the richness and accuracy of the MSA data they utilized. Recently, AlphaFold3 [24] has been meticulously developed and boasts remarkable capabilities in reconstructing the structure of complex biomolecular assemblies, including amino acids, nucleic acids, small molecules and others. In structural biology, this is a significant breakthrough, offering scientists unprecedented insights into the complex interactions and architectures of biological molecules. A modification of AlphaFold2’s architecture, AlphaFold3 uses only four blocks to interact with MSA information and pair representations, reducing dependence on MSA. Generated AI models are used to generate protein structure modules based on these learned representations. Furthermore, AlphaFold3 incorporates a diffusion module that estimates raw atomic coordinates directly, which works with amino-acid-specific frames and torsion angles for side chains. It eliminates the steps involved with traditional frame- and angle-based representations, making it possible to predict molecular structures more directly and holistically. In this process, the stages of bioinformatics FMs are outlined from the earliest presentations to current versions.

## BIOINFORMATICS FM IN GENOMICS

Using Transformer to decode DNA’s language has gained attention for deciphering biological functions through universal genetic codes, which explain DNA’s translation into proteins. DNABERT [20] captures global and transferrable insights into genomic DNA sequences by using a transformer. Using the Nucleotide Transformer [25], foundational language models can be built and pre-trained across genomic datasets. In DNABERT-2 [26], byte pair encodings are modified for improved computational efficiency, and input length constraints are addressed using multiple strategies. To adapt to novel tasks, HyenaDNA [27] leverages longer context length and a sequence length scheduling technique. From the molecular level to genomic scale, Evo [28] is a long-context foundation model that facilitates both predictive and generative tasks. VQDNA [29] has redefined genome tokenization into a holistic system based on data patterns using VQ-VAE (Vector Quantized Variational Autoencoder) for learning genome vocabulary. Pre-trained FMs have been trained on multi-species datasets, and used to predict promoters, enhancers, transcription factor binding sites and cis-regulatory elements. Bioinformatics FMs in genomics are reported in Table 1. The long sequence length of biological sequences presents many challenges during the training process, making these models unable to address some biological problems. As large-scale sequence modeling, advances in biology and genomics have been rapid. Caduceus [30] represents the inaugural reverse complementarity (RC) equivariant bi-directional long-range DNA foundation model, demonstrating superior performance to its predecessors in the realm of long-range models.

**Table 1.** Bioinformatics FMs in genomics.

Model	Architecture	Description
Language FMs		
**DNABERT** [21]	$\bullet$ Transformer (encoder only)	- Promoter region prediction
	$\bullet$ Pre-train + fine-tuning	- TF binding site identification
	$\bullet$ 110 million parameters	- Canonical splice site recognition
		- Functional genetic variant identification
**iDNA-ABT** [36]	$\bullet$ Transformer (encoder only)	- 4mC and non-4mC
	$\bullet$ No pre-train	- 5hmC and non-5hmC
	$\bullet$ 1.6 million parameters	- 6mA and non-6mA
**iDNA-ABF** [37]	$\bullet$ Transformer (encoder only)	- 4mC and non-4mC
	$\bullet$ Pre-train + fine-tuning	- 5mC and non-5mC
	$\bullet$ 110 million parameters	- 6mA and non-6mA
**DNABERT-2** [26]	$\bullet$ Transformer (encoder only)	- Core/proximal promoter prediction
	$\bullet$ Pre-train + fine-tuning	- Epigenetic mark prediction
	$\bullet$ 117 million parameters	- TF binding site prediction
		- Splice site prediction
**Nucleotide**	$\bullet$ Transformer (encoder only)	- Enhancer prediction
**transformer** [25]	$\bullet$ Pre-train + fine-tuning	- Promoter prediction
	$\bullet$ 500 million parameters	- Epigenetic mark prediction
		- Splice site prediction
**HyenaDNA** [27]	$\bullet$ Hyena (decoder only)	- Enhancer prediction
	$\bullet$ Pre-train + fine-tuning	- Promoter prediction
	$\bullet$ 1.6 million parameters	- Epigenetic mark prediction
		- Splice site prediction
**Evo** [28]	$\bullet$ StripedHyena (decoder only)	- Fitness effect prediction
	$\bullet$ Pre-train + fine-tuning	- Essential gene prediction
	$\bullet$ 7 billion parameters	- Prokaryotic promoter-RBS pairs
Vision FMs		
**VQDNA** [29]	$\bullet$ VQ-VAE	- Promoter detection
	$\bullet$ Pre-train + fine-tuning	- Core promoter detection
	$\bullet$ 110 million parameters	- Splice site prediction
		- TF binding site prediction
Multimodal FMs		
**Enformer** [33]	$\bullet$ CNN + Transformer	- Activating/repressive mutations
	$\bullet$ No pre-train	- Conserved and non-conserved enhancers
	$\bullet$ 228 million parameters	- Arbitrary sequence predictions
**GPN** [32]	$\bullet$ CNN + Transformer	- Genome-wide variant effects
	$\bullet$ Pre-train + fine-tuning	- Genome structure
	$\bullet$ 65 million parameters	- DNA motifs
**DeepSEED** [35]	$\bullet$ cGAN + DenseNet-LSTM	- DNA sequence generation
	$\bullet$ Pre-train + fine-tuning	- Constitutive promoter design
	$\bullet$ 130 million parameters	- Dox-inducible promoter design
		- IPTG-inducible promoter design

### Genome-wide variant effect prediction

Mutations in DNA sequences play a significant role in contributing to species diversity. A genome-wide association study (GWAS) provides an essential amount of biological insight across a wide range of species. AI architectures have evolved to accommodate the complexity of genomic data and the nuances of high-dimensional modalities available for measuring the genome. DeepSEA [31] learns non-coding variant effects on the DNA sequence alone, outperforming supervised deep learning models. In the last decade, CNN has dominated deep learning models of the DNA sequence. A genomic pre-trained network (GPN) [32] investigates effects of genome-wide variants by training models on DNA sequences. Unlike conventional GWAS methods, GPN demonstrates exceptional proficiency in forecasting the impact of rare variants. A number of foundational DNA sequence language models, including DNABERT, DNABERT-2 and Nucleotide Transformer, also predict variants from DNA sequences. Collectively, these advances enhance our understanding of how DNA sequence mutations produce biological diversity.

### DNA cis-regulatory region prediction

In the regulation of gene expression, cis-regulatory sequences, including enhancers and promoters, play a pivotal role and contribute to the design of tissue-specific elements. To comprehend their functions and their associations with diseases, identifying these sequences in DNA is an essential challenge. Enformer [33] predicts gene expression and promoter-enhancer interactions by utilizing a large receptive field, for identifying cis-regulatory regions and offering valuable insights into their functions. A new transfer learning approach based on DNABERT, iEnhancer-BERT [34], facilitates enhancer prediction by utilizing an innovative DNABERT algorithm. In contrast to conventional fine-tuning methods, iEnhancer-BERT applies a CNN layer to classify output from the transformer encoder layers. Thus, biological sequences are now being recognized as the natural language of computational modeling. Furthermore, DeepSEED [35] integrates expert knowledge with learning methodologies to design synthesized promoters that are effective for synthetic promotion.

### DNA methylation identification

A fundamental biological process is DNA methylation, which regulates gene expression epigenetically. Various medical conditions are associated with this process, which can also serve as a marker for metagenomic binning. AI models have advanced our understanding of DNA methylation in a variety of biological processes. Currently, iDNA-ABT [36], iDNA-ABF [37] and ccsmeth [38] serve as versatile predictors for a range of methylation predictions, including 6-methyadenine (6mA), 5-hydroxymethylcytosine (5hmC) and 4-methylcytosine (4mC). In iDNA-ABT, transductive information maximization is used in conjunction with adaptive embedding, but its potential for detecting DNA methylation patterns is yet to be explored. iDNA-ABF employs a multi-scale architecture instead of a single tokenizer. Based on tokenization, BERT encoders are able to extract diverse embeddings to produce the final evolutionary output. Furthermore, ccsmeth detects haplotype-aware methylation using nanopore sequencing data and PacBio Circular Consensus Sequencing (CCS) data, and makes use of the symmetry and aggregation characteristics of 5mC sites for prediction.

## BIOINFORMATICS FM IN TRANSCRIPTOMICS

The advancement of BERT-based language models tailored for RNA sequences exhibiting reduced conservation has facilitated the emergence of significant RNA foundation models, such as RNA-FM [39] and RNA-MSM [40]. RNA-FM predicts 2D/3D structures based on self-supervised learning, capturing a variety of structural information that provides a comprehensive understanding of RNA sequence features. RNA-MSM utilizes homologous sequences from RNAcmap, which excels at mapping base pairing probabilities and solvent accessibility to 2D base pairing probabilities. Furthermore, several RNA generative models have recently been proposed by generative AI technology, such as RfamGen [41] and GenerRNA [42]. In RfamGen, alignment information and consensus secondary structure data are explicitly integrated into deep generative models to facilitate the design of RNA family sequences. GenerRNA represents a large-scale model that can be employed for the automation of RNA design. Various RNA sequence, structure and function tasks can be fine-tuned using PTMs. Bioinformatics FMs in transcriptomics are reported in Table 2.

**Table 2.** Bioinformatics FMs in transcriptomics.

Model	Architecture	Description
Language FMs		
**RNABERT** [43]	$\bullet$ Transformer	- RNA family classification
	$\bullet$ Pre-train + fine-tune	- Novel transcript annotation
		- RNA secondary structure prediction
**RNA-FM** [39]	$\bullet$ Transformer	- Gene expression regulation
	$\bullet$ Pre-train + fine-tune	- SARS-CoV-2 genome evolution
	$\bullet$ 23 million parameters	- RNA secondary structure prediction
**SpliceBERT** [44]	$\bullet$ Transformer	- Variant effects on splicing
	$\bullet$ Pre-train + fine-tune	- Cross-species splice site prediction
	$\bullet$ 19.4 million parameters	- Human genome branch point prediction
**RNA-MSM** [40]	$\bullet$ Transformer	- RNA solvent accessibility
	$\bullet$ Pre-train + fine-tune	- RNA secondary structure prediction
**GenerRNA** [42]	$\bullet$ Transformer (decoder only)	- De novo RNA generation
	$\bullet$ Pre-train + fine-tune	- RNA generation with specific properties
Vision FMs		
**RfamGen** [41]	$\bullet$ VAE + covariance model	- Functional RNA family generation
	$\bullet$ No pre-train	- RNA family sequence representation
Multimodal FMs		
**Bert2Ome** [46]	$\bullet$ CNN + Transformer	- 2-O-methylation site prediction
	$\bullet$ Pre-train + fine-tune	
	$\bullet$ 110 million parameters	

### RNA secondary structure prediction

In molecular biology, RNA secondary structure prediction is a significant challenge that requires improving structure prediction models and better understanding RNA folding. A transformer model, tokens and position embeddings, and pre-trained tasks are all integral parts of RNABERT [43]. RNABERT predicts secondary structures, classifies RNA families and annotates uncharacterized transcripts, thereby elucidating RNA structural properties.

### RNA splice site prediction

Eukaryotic organisms rely on RNA splicing for post-transcriptional gene expression. Through the development of PTMs known as SpliceBERT [44], researchers have made significant advances in sequence-based modeling of RNA splicing. Apart from capturing RNA splicing dynamics, SpliceBERT also enables the identification of splice-disrupting variants, which can be prioritized according to their impact on the output. Therefore, researchers are able to gain insight into genetic variations influencing RNA splicing, facilitating effective identification and prioritization of potentially significant variations.

### RNA modification detection

Biological processes rely on modifications to RNA during post-transcription. In gene expression regulation, N7-methylguanosine (m7G) and 2’-O-methylation (Nm) RNA modifications represent widespread post-transcriptional modifications in various cellular processes. Using transformer architecture and stacking ensemble techniques, BERT-m7G [45] is a transformative computational tool for precisely pinpointing m7G sites, which is advantageous over labor-intensive experimental approaches. BERT-m7G enables us to uncover post-transcriptional modifications and better understand how m7G affects gene expression. Bert2Ome [46] provides profound insights into the underlying biological mechanisms by directly inferring 2’-O-methylation modification sites. Bert2Ome uses an integrated BERT-based model and CNN to investigate intricate relationships between modifications and RNA sequence content.

## BIOINFORMATICS FM IN PROTEOMICS

Proteins play a pivotal role in constructing and maintaining vital processes in life. Protein research has experienced a substantial surge in data accumulation as the field has advanced. Structures of proteins determine how they interact with other molecules and how they function. LLMs provide an effective means of extracting pertinent and valuable information from extensive datasets. ProteinBERT [47] excels at predicting major post-translational modifications, which can be attributed to the incorporation of GO annotation prediction tasks. ProteinBERT has outperformed other deep learning models with larger parameters on various benchmarks covering diverse protein properties. The earliest protein pre-trained method that integrates external knowledge graphs is OntoProtein [48]. Aside from inheriting the strong ability of pre-trained protein language models, the knowledge embedding object also extracts biology knowledge from the knowledge graph. OntoProtein uses generative models to streamline protein downstream tasks. Bioinformatics FMs in proteomics are reported in Table 3. As part of the evaluation of deep learning models in protein science [49], numerous applications and performance characteristics of proteomics FMs are presented, including protein structure classification and enzyme function prediction. Additionally, Critical Assessment of Structure Prediction (CASP) aims to objectively test the structure prediction methods of research groups from around the world. It is feasible for CASPers to evaluate where future efforts could be most effectively directed by categorizing various themes.

**Table 3.** Bioinformatics FMs in proteomics.

Model	Architecture	Description
Language FMs		
**UniRep** [59]	$\bullet$ mLSTM	- Protein engineering
	$\bullet$ Pre-train + fine-tune	- Remote homology detection
	$\bullet$ 18.2 million parameters	- Mutation effect identification
**MSA Transformer** [50]	$\bullet$ Transformer	- Contact prediction
	$\bullet$ Pre-train + fine-tune	- Secondary structure prediction
	$\bullet$ 100 million parameters	
**ProtTrans** [51]	$\bullet$ Transformer	- Protein subcellular localization
	$\bullet$ Pre-train + fine-tune	- Secondary structure prediction
	$\bullet$ 11 billion parameters	
**ProteinBERT** [47]	$\bullet$ Transformer	- Evolutionary: remote homology
	$\bullet$ Pre-train + fine-tune	- Engineering: fluorescence, stability
	$\bullet$ 16 million parameters	- Secondary structure prediction
**ProtGPT2** [55]	$\bullet$ Autoregressive transformer	- Protein sequence generation
	$\bullet$ Pre-train + fine-tune	
	$\bullet$ 738 million parameters	
**ZymCTRL** [56]	$\bullet$ Transformer	- Enzyme generation
	$\bullet$ Pre-train + fine-tune	
	$\bullet$ 700 million parameters	
**ESM2** [53]	$\bullet$ Transformer	- Contact prediction
	$\bullet$ Pre-train + fine-tune	- Protein-protein interactions
	$\bullet$ 15 billion parameters	- Evolutionary: remote homology
		- Engineering: fluorescence, stability
		- Secondary structure prediction
**ProGen** [57]	$\bullet$ Autoregressive Transformer	- Protein sequence design
	$\bullet$ Pre-train + fine-tune	
	$\bullet$ 1.2 billion parameters	
Multimodal FMs		
**OntoProtein** [48]	$\bullet$ BERT + knowledge graph	- Contact prediction
	$\bullet$ Pre-train + fine-tune	- Protein-protein interactions
		- Evolutionary: remote homology
		- Engineering: fluorescence, stability
		- Protein function prediction: GO
		- Secondary structure prediction
**AlphaFold3** [24]	$\bullet$ Diffusion	- Protein-ligand interactions
	$\bullet$ No Pre-train	- Protein-nucleic acid interactions
		- Antibody-antigen prediction
		- Protein structure prediction

### Protein structure prediction

Functionality and interaction of proteins are closely related to their structure. Deep learning has gradually improved prediction accuracy and computational speed in predicting protein structures. MSA Transformer [50] constructs a protein language model from MSA. Masked language model (MLM) objectives are used to build the model across many protein families. According to the experience with BERT, when it comes to predicting secondary structure or contact, it appears that a model with more parameters is easier to use. ProtTrans [51] appears to be the only model with more parameters than most other models. Furthermore, ProtTrans has made tremendous progress in the prediction of per-residue structure. TAPE [52] establishes a standardized evaluation system for protein transfer learning. Five distinct problems are included in the task set, including protein structure prediction, fluorescence landscape prediction, stability landscape prediction and protein design. With up to 15 billion parameters, ESM2 [53] trained transformer protein language models for widespread protein downstream applications. A protein structure predictor, ESMFold, developed later by the ESM2 team, demonstrates accuracy that is nearly comparable to alignment-based approaches, while significantly improving processing speed. As the model was scaled up, insights regarding the atomic level structure began to emerge. PeSTo [54] is a parameter-free geometric deep learning approach designed to identify proteins binding to others. Recently, AlphaFold3 [24] has been developed and can accurately predict protein complexes with less emphasis on co-evolutionary information.

### Protein sequence generation

Protein generation is widely applied to drug development and protein engineering. In order to form stable three-dimensional structures, it is hoped that the generated sequences may have good foldability. In addition, it is expected that the desired proteins have specific functional properties, such as enzyme activity. In the field of protein generation, the advancement of LLMs and the incorporation of conditional models has significantly progressed. With ProtGPT2 [55], protein amino acid propensities are generated according to natural principles, modeled after the impressive achievements of transformer-based language models. Several globular characteristics that correspond to natural proteins are observed in ProtGPT2-generated proteins, according to analyses involving disorder and secondary structure prediction. The ZymCTRL language model [56] generates artificial enzymes conditionally upon prompts from the Enzyme Commission. Generated sequences are globular, ordered and distanced from known protein spaces, and they perform their intended functions. A new algorithm, ProGen [57], integrates UniprotKB keywords into conditional tags and generates proteins with desired structural properties.

### Protein evolution and mutation detection

Protein sequences and structures undergo changes during biological evolution. In order to produce functional diversity in proteins, evolution and mutation play a vital role. It has been suggested that protein language models can effectively predict evolutionary changes and mutations. With DeepSequence [58], a probabilistic model is learned across protein families, and it is superior to existing methods that use evolutionary data to predict mutation effects. It captures conservation in biological data and uses an evidence lower bound to score mutations. A new model, UniRep [59], is developed using long short-term memory (LSTM) to detect remote homologies and mutation effects. EVOLVEpro [60] outperforms existing methodologies, achieving improvements of up to 100-fold in targeted properties across six proteins within the domains of RNA production, genome editing and antibody binding applications. These findings underscore the benefits of few-shot active learning with minimal experimental data compared to zero-shot predictions.

## BIOINFORMATICS FM IN DRUG DISCOVERY

For computer-aided drug discovery, expert knowledge algorithms are used to screen drug molecules, their lead compounds and their interactions with target molecules. A new approach to molecular fingerprinting, SMILES-BERT [61], departs from knowledge-based molecular fingerprints as input. To represent molecules, SMILES sequences are encoded based on a BERT-based model. Compared to previous models reliant on molecular fingerprints, this approach produced superior results across multiple downstream predictions of molecular properties. Through the Baidu PaddlePaddle platform, X-MOL [62] uses a pre-training model for molecular understanding of SMILES, fine-tuning downstream molecular analysis tasks, such as predicting molecular properties, analyzing chemical reactions, predicting drug-drug interactions and optimizing molecules. Bioinformatics FMs in drug discovery are reported in Table 4. For the evaluation of drug FMs, ADMETlab 2.0 [63] has been developed as a web-based system for ADMET, enhancing early drug-likeness evaluation and accelerating drug discovery. A total of 288 967 entries are contained in the ADMET database, where four functions are available for users to easily analyze six types of drug likeness, predict 31 ADMET endpoints and perform systematic evaluations and database/similarity searching. Various aspects of physicochemical, medicinal and ADME properties, as well as toxicity endpoints and toxicophore rules, are evaluated by ADMET for drug discovery. These metrics include 17 physicochemical properties, 13 medicinal chemistry properties, 23 ADME properties and 8 toxicophore rules.

**Table 4.** Bioinformatics FMs in drug discovery.

Model	Architecture	Description
Language FMs		
**SMILES-BERT** [61]	$\bullet$ BERT	- Molecular representation
	$\bullet$ Pre-train + fine-tune	- Molecular property prediction
**MolGPT** [69]	$\bullet$ Transformer (decoder only)	- Molecule generation via properties
	$\bullet$ Pre-train + fine-tune	- Molecule generation via scaffolds
	$\bullet$ 6 million parameters	
**X-MOL** [62]	$\bullet$ Transformer	- Molecular property prediction
	$\bullet$ Pre-train + fine-tune	- Chemical reaction analysis
		- Molecule optimization
**K-BERT** [64]	$\bullet$ BERT	- Atom feature prediction
	$\bullet$ Pre-train + fine-tune	- Molecular feature prediction
	$\bullet$ 110 million parameters	
**DrugBAN** [73]	$\bullet$ FCS	- Drug-target pair prediction
	$\bullet$ No Pre-train	
Vision FMs		
**PMDM** [71]	$\bullet$ EGNNs + SchNet	- Molecule generation of specific target
	$\bullet$ No pre-train	
**POLYGON** [72]	$\bullet$ VAE	- Molecule generation of multi-target
	$\bullet$ No pre-train	
Graph FMs		
**Mole-BERT** [65]	$\bullet$ GINs	- Molecular property prediction
	$\bullet$ Pre-train + fine-tune	- Drug-target affinity prediction
**MolCLR** [67]	$\bullet$ GNN + contrastive learning	- Molecular representation
	$\bullet$ Pre-train + fine-tune	- Molecular property prediction
**Pocket2Mol** [70]	$\bullet$ MPNN	- Molecule generation via 3D pockets
	$\bullet$ No pre-train	
**KPGT** [66]	$\bullet$ Line Graph Transformer	- Molecular representation
	$\bullet$ Pre-train + fine-tune	- Molecular property prediction
	$\bullet$ 100 million parameters	
**EIHGN** [74]	$\bullet$ 3D GNN	- Protein-ligand binding prediction
	$\bullet$ No pre-train	
Multimodal FMs		
**MoleculesSTM** [68]	$\bullet$ MolBART + GIN + SciBERT	- Structure-text retrieval
	$\bullet$ Pre-train + fine-tune	- Molecule editing
	$\bullet$ 120 million parameters	

### Drug-like molecular property prediction

In drug discovery, PTMs determine molecular properties in downstream tasks, such as absorption, distribution, metabolism, excretion and toxicology (ADMET) and pharmacokinetics (PK). K-BERT [64] differs from BERT by adopting three distinct pre-trained tasks as part of its pre-training phase, which goes beyond mere discovery of the SMILES paradigm to understand its essence. Masked atom modeling and triplet masked contrastive learning tasks are introduced in Mole-BERT [65], a graph-based pre-training neural network based on BERT. A network can acquire a comprehensive understanding of molecular graph ‘language’ through these tasks. Using self-supervised learning, KPGT [66] is pre-trained for the Line Graph Transformer. The molecular graph is processed into a molecular line graph, and molecular fingerprints are used as additional knowledge, resulting in better prediction ability in downstream tasks such as molecular property prediction. To train molecular property prediction models, researchers have gradually adopted the large model + contrast learning paradigm due to the increasing prominence of contrastive learning. In MolCLR [67], a contrastive learning pre-training architecture, the data from one molecular graph before and after enhancement are treated as a positive sample, while data from different molecular graphs are considered a negative sample. MoleculesSTM [68] constructs a multimodal molecular text pre-training model with two branches for molecular prediction, which reduces the representation distance between chemical structure and text description.

### Drug-like molecule generation

Virtual screening libraries usually contain a few compounds, not entire drug-like chemicals. As part of MolGPT [69], an additional training task is included to facilitate conditional prediction. As well as being capable of generating innovative and effective molecules, the model also captures specific statistical characteristics within the dataset. Recently, researchers have introduced target protein information into molecular generation to identify potential target molecules. In Pocket2Mol [70], chemical constraints are captured through an E(3)-equivariant generative model. Using a neural network architecture with E(3) equivariant, protein pockets and molecular fragments can be extracted more accurately. Using a conditional deep generative model, PMDM [71] can efficiently generate 3D molecules that are highly affinable to specific proteins. In order to preserve the geometric properties of molecules, the system uses a dual diffusion strategy that captures both local and global interactions between atoms as well as a dynamic kernel that is equivariant. Researchers are also gradually studying multi-target molecules in addition to single-target molecules. A deep generative model, POLYGON [72], can design new polypharmacology compounds that inhibit multiple targets simultaneously using encoder-decoder architectures and reinforcement learning strategies.

### Drug-target interaction identification

Drug-target interaction provides valuable guidance for optimizing pharmaceutical agents. DrugBAN [73] uses frequent contiguous subsequence (FCS) mining to extract high-quality substructures of targets and drugs. To explicitly learn drug-target interactions, it then constructs a bilinear attention network framework. To enhance the generalization to novel drug-target pairs, a conditional domain adversarial network is used to harmonize the interaction representations across various domains. In EIHGN [74], four independent GNNs are used to learn node representations from four distinct atomic interactions to model complexes as heterogeneous graphs. As a result, the risk of overshadowing non-covalent interaction information during message passing is minimized. EIHGN also decomposes affinity prediction values into the sum of non-covalent interaction forces predicted between target and drug atoms.

## BIOINFORMATICS FM IN SINGLE-CELL ANALYSIS

Single-cell RNA sequencing (scRNA-seq) technology has paved the way for numerous breakthroughs. Single-cell language models can be used to identify cell states, discover novel cell types, infer regulation networks and integrate multi-omics data. scGPT [75] provides a unified pre-training pipeline tailored to non-sequential datasets. Through the use of stacked transformer layers and multiple heads, scGPT is capable of general-purpose pre-training and fine-tuning for specific applications, enabling learning to be transferred to downstream tasks. To infer the missing single-cell proteome from the transcriptome, scTranslator [76] proposes a large, pre-trained, generative model derived from both natural language processing (NLP) and genetic central dogma. In scTranslator, the protein abundance is inferred from paired bulk data, then paired single-cell data and finally from scRNA-seq datasets as a transformer-based model. scMVP [77] handles sequencing data that simultaneously measure gene expression and chromatin accessibility in the same cell, which can help mitigate data sparsity issues with imputation and identify cell groups for different joint profiling techniques. scButterfly [78] learns latent factors within individual modalities to perform cross-modal translation using a dual aligned variational autoencoder and data augmentation scheme. A masked VAE is trained by scButterfly, then the latent representations are cross-modally aligned. The scFoundation [79] algorithm presents a novel pre-trained method called read-depth-aware modeling. Nicheformer [80] is a transformer-based approach to learning cellular representations from dissociated single cells and transcriptomics data for many downstream applications. CELLama [81] creates cellular data embedding sentences encapsulating gene expressions and metadata. Bioinformatics FMs in single-cell multi-omics analysis are reported in Table 5. In order to evaluate single-cell foundation models, scEval [82] evaluates the hyperparameters and LLM training, which presents a summary of single-cell LLMs and their limitations, as well as possible future developments. Several single-cell LLMs were evaluated on eight tasks with 22 datasets.

**Table 5.** Bioinformatics FMs in single-cell analysis.

Model	Architecture	Description
Language FMs		
**scBERT** [86]	$\bullet$ BERT	- Gene-gene interaction prediction
	$\bullet$ Pre-train + fine-tuning	- Cell type annotation
		- Novel cell type discovery
**scMVP** [77]	$\bullet$ Transformer	- Data imputation
	$\bullet$ No pre-train	- Cell group identification
**scTranslator** [76]	$\bullet$ Transformer	- Gene-gene interaction prediction
	$\bullet$ Pre-train + fine-tuning	- Gene pseudo-knockout
		- Cell clustering
**TOSICA** [85]	$\bullet$ Transformer	- Cell type annotation
	$\bullet$ No pre-train	
**scFoundation** [79]	$\bullet$ Transformer	- Gene expression enhancement
	$\bullet$ Pre-train + fine-tuning	- Single-cell drug response prediction
	$\bullet$ 100 million parameters	- Tissue drug response identification
**scGPT** [75]	$\bullet$ Transformer	- Cell clustering
	$\bullet$ Pre-train + fine-tuning	- Batch correction
		- Gene regulatory network inference
**mvTCR** [88]	$\bullet$ Transformer	- Cell-level embedding
	$\bullet$ No pre-train	- Atlas-level analysis
Vision FMs		
**scButterfly** [78]	$\bullet$ VAE	- Cell type annotation
	$\bullet$ Pre-train + fine-tuning	- Poor-quality data enhancement
		- Integrative multi-omics analysis
**MIDAS** [90]	$\bullet$ VAE + transfer learning	- Modality alignment
	$\bullet$ No pre-train	- Data imputation
		- Batch correction
Graph FMs		
**DeepMAPS** [87]	$\bullet$ Graph Transformer	- Cell clustering
	$\bullet$ No pre-train	- Biological network construction
**SiGra** [89]	$\bullet$ Graph Transformer	- Spatial profile augmentation
	$\bullet$ No pre-train	
**MarsGT** [83]	$\bullet$ Graph Transformer	- Cell clustering
	$\bullet$ No pre-train	- Gene regulatory network inference
**scPROTEIN** [84]	$\bullet$ GCN + contrastive learning	- Cell type annotation
	$\bullet$ No pre-train	- Batch correction
		- Cell type annotation
		- Proteomic data exploration
Multimodal FMs		
**GLUE** [22]	$\bullet$ Graph VAE	- Triple-omics data integration
	$\bullet$ No pre-train	- Integrative regulation inference

### Cell clustering

In order to understand the complex landscape of cellular heterogeneity within biological samples, the process of cell clustering is crucial. To learn cell embedding for clustering, the encoder and decoder structures of scFoundation [79] are transformer-based models, and only genes that are not masked are fed into the encoder. MarsGT [83] infers and identifies rare cell clusters from multi-omics data generated by single cells. MarsGT builds a multi-head attention mechanism on heterogeneous graphs of genes and cells. In scPROTEIN [84], peptide quantification uncertainty and other data problems are addressed in a unified framework through deep graph contrastive learning. A variety of downstream tasks can be performed using scPROTEIN’s versatile cell embeddings.

### Cell type annotation

When annotating a single cell, biological labels are assigned to each cell or cluster, typically the cell type or cell state. With the remarkable success of LLMs in NLP and CV, single-cell RNA sequencing data can now be analyzed for cell type annotation. Several computational tools have emerged for annotation of scRNA-seq data using language models, including TOSICA [85] and scBERT [86]. TOSICA incorporates knowledge-based masks from GSEA into a fully connected weight matrix to create an interpretable cell type annotation method. The pre-training phase of scBERT is designed to eliminate batch effects and enhance generalizability through a comprehensive understanding of gene-gene interactions. When fine-tuning, reference datasets influence model parameters since a classifier is added to PTMs. Thus, scBERT enables the discovery of unbiased long-range interactions and data-driven annotation of cell types.

### Multi-omics integration

Integration of various omics technologies offers several advantages over analyses based on individual omics data. Large models are valuable tools for finding solutions to scMulti-omics data’s feature variance, sparsity and cell heterogeneity because of their adaptability, generalization capabilities and feature extraction abilities. As part of scMulti-omics integration tasks, scGPT [75] uses supplementary token sets to signify distinct sequencing modalities. Transformators incorporate modality tokens, either at the feature or cell level, into their output. Incorporating this intentionally prevents the transformer from highlighting features associated with the same modalities, while simultaneously undermining those associated with different modalities. In DeepMAPS [87], scMulti-omics data are integrated and mapped into biological networks using a graph transformer. Because of the fact that DeepMAPS builds a graph with nodes for genes and cells, the features of all other modes are mapped to genes. The transformer in DeepMAPS builds relations between cells and genes as well as gene-gene relations using local and global features. A cell-level embedding is created using mvTCR [88], which is easily scalable to atlas-level analysis and fits well into a standard analysis pipeline. Using separate encoders, mvTCR combines different modalities to produce a joint representation. Using an image-augmented graph transformer, SiGra [89] reveals single-cell spatial information. Through the use of multimodalities and transcriptomics, SiGra enhances data quality and recognizes spatial domains simultaneously. GLUE [22] integrates unpaired multi-omics data and infers regulatory interactions. GLUE models cross-layer regulatory interactions explicitly by leveraging prior biological knowledge. As well as integrating triple omics, GLUE can also handle regulating inference and annotation correction. MIDAS [90] uses a modular encoder network and a decoder network to integrate and transfer multimodal data from single cells.

## DATA IN THE BIOINFORMATICS FM

In bioinformatics, FMs are reliant on biological data quality, which constitutes a massive amount of multi-omics data. Biological databases are commonly used in bioinformatics FMs, as shown in Table 6. In genomics, The Cancer Genome Atlas (TCGA) [91] analyzes more than 20 000 cancer samples matched to normal samples, covering 33 different cancer types; TargetFinder [92] provides a pipeline for identifying or characterizing gene targets of distal enhancers; ArrayExpress [93] contains data from high-throughput functional genomics experiments. In transcriptomics, Gene Expression Omnibus (GEO) [94] archives functional genomics data derived from microarrays and other high-throughput methodologies; Encyclopedia of DNA Elements (ENCODE) [95] is a comprehensive database of essential elements in the human genome, including proteins and RNA, as well as regulatory elements that control cell activity and gene expression. In proteomics, Universal Protein Resource (UniProt) [96] contains comprehensive protein sequences and annotations; Protein Data Bank (PDB) [97] contains sequences and 2D/3D structures of large biological molecules. In drug discovery, ChEMBL [98] is a meticulously curated database of bioactive molecules exhibiting drug-like properties; ZINC [99] is a publicly accessible database of commercially available compounds designed for virtual screening; PubChem [100] is the collection of chemical information that is freely accessible. In single-cell data, Single-cell Expression Atlas [101] serves as a comprehensive database for single-cell gene expression across various species; Human Cell Atlas [102] aims to map each cell type within the human body, thereby creating a 3D atlas of human cells.

**Table 6.** Biological databases commonly used in bioinformatics FMs.

Database	Description	Web link
Genomics		
**TCGA** *2.5 petabytes*	Database of cancer genome atlas varieties	https://www.cancer.gov/ccg/research/genome-sequencing/tcga
**TargetFinder** *100 thousand*	Database of promoter-enhancer interaction pairs	https://github.com/shwhalen/targetfinder
**ArrayExpress** *78 thousand*	Database of the high-throughput functional genomics data	https://www.ebi.ac.uk/biostudies/arrayexpres
Transcriptomics		
**GEO** *7.3 billion*	Database of public functional genomics data	https://www.ncbi.nlm.nih.gov/geo/
**ENCODE** *100 million*	Database of functional elements in the human genome	https://www.encodeproject.org/
Proteomics		
**UniProt** *245 million*	Database of protein sequences and functions	https://www.uniprot.org/
**PDB** *222 thousand*	Database of 3D structural data for large biological molecules	https://www.rcsb.org/
Drug		
**ChEMBL** *2.4 million*	Database of bioactive compounds with drug-like properties	https://www.ebi.ac.uk/chembl/
**ZINC** *750 million*	Database of commercially available compounds	https://zinc15.docking.org
**PubChem** *119 million*	Database of freely available chemical information	https://pubchem.ncbi.nlm.nih.gov/
Single cell		
**Single-Cell Expression Atlas** *10 million*	Database of single-cell gene expression across species	https://www.ebi.ac.uk/gxa/sc/home
**Human Cell Atlas** *61.8 million*	Database of the multi-omic human cell atlas	https://www.humancellatlas.org/

## FUTURE DIRECTIONS

**Figure 3.**
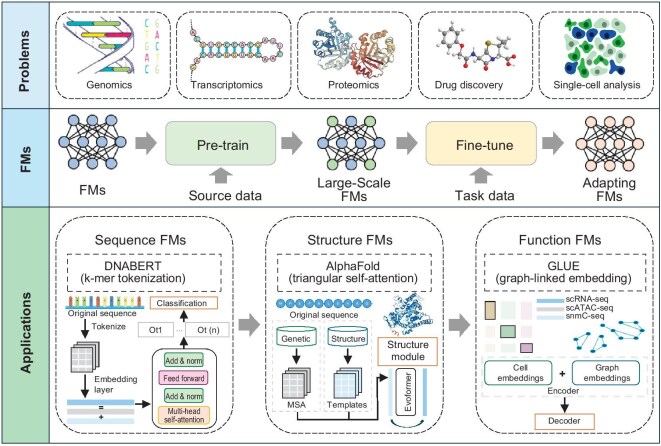
Landscape of bioinformatics FMs.

Our study focuses on various applications of bioinformatics FMs, which accurately model the intricate complexities of molecular biology. Pre-training architectures capture patterns related to source data; fine-tuning strategies analyze task data to solve biological problems accurately. The landscape of FMs in bioinformatics is shown in Fig. 3. New insights can be gained into the dynamic interaction between molecules by exploring these cutting-edge technologies. Our final objective is to discuss challenges and opportunities related to explainability of foundation models, and architectures of large-scale models.

### Pre-training paradigm

The emerging methodologies for training foundation models in artificial intelligence enable the execution of specific downstream tasks, thereby allowing AI to be fine-tuned for highly specialized applications even when only a limited number of training examples are available. Currently, several studies are exploring the application of prompt learning and contrastive learning to pre-trained models within the field of bioinformatics, which require further development. Prompt learning and contrastive learning have become pivotal techniques in bioinformatics, particularly when utilized with pre-trained models to enhance both model performance and interpretability across various bioinformatics tasks. KANO [103] exploits external fundamental domain knowledge to enhance molecular contrastive learning and fine-tune learning. Microscopic atomic associations are investigated while maintaining the molecular semantics of an element-oriented knowledge graph. In fine-tuning, functional prompts are designed to elicit task-specific knowledge. PromptProtein [104] offers an innovative pre-training and fine-tuning framework based on prompt-guided training. With prompt-guided multi-task pre-training, it learns to focus on different structure levels based on multiple prompt signals. By providing downstream tasks with on-demand flexibility, prompt fine-tuning modules allow them to utilize respective levels of structure information.

### Evaluation framework

Several AI models have been trained on large datasets and applied to downstream applications. Foundation models offer opportunities and risks from their capabilities and technical principles to their applications and societal impacts. The scale and effectiveness of models across so many tasks encourage homogenization. Using three key components for model evaluation (models, data and metrics), UltraEval [105] showcases itself as a user-friendly evaluation model that is lightweight, comprehensive, modular and efficient. Some studies have also evaluated the performance of some bioinformatics research fields, including protein engineering, drug design and analysis of single-cell multi-omics data. scBackdoor [106] has been introduced for single-cell pre-training models to assess the attack success rate. This poses a significant potential threat to single-cell research, particularly in relation to AI pre-training models that rely on open data.

### Model explainability

Bioinformatics also faces challenges in providing interpretable FMs and acquiring logical evidence. For instance, computer-aided drug discovery includes docking, scoring and screening. To generate a drug molecule, properties such as effectiveness, novelty and similarity to existing drugs must be considered. However, existing methods lack extensive studies on actual chemical or biological experimental validation to prove their efficiency. FMs may be able to solve complex biological problems more efficiently through knowledge graphs with interpretability and explainability. The use of causal inference has been shown to enhance predictive accuracy, fairness, robustness and explainability of NLP models by tracing causal relationships among variables. With its improved sampling efficiency, CIMI [107] provides more faithful and generalizable explanations, making it particularly suitable for large pre-trained models.

### Hallucination detection

Foundation models are employed to construct comprehensive biological maps in such a diverse environment. However, there are difficulties transitioning from derivable approaches to multi-focus frameworks. For example, large language models are universal tools for a broad range of biological datasets and applications. In order to improve understanding of the cellular landscape, various model architectures are synergized and scaled up to extract meaningful features from raw data. Furthermore, LLMs can reason and answer questions impressively, but they tend to hallucinate false results and answer questions unsubstantiated [108]. Hallucination in FMs refers to the generation of content that strays from factual reality or includes fabricated information. Current hallucination detection techniques lack accuracy, low latency and low cost all at once. Luna [109] has been fine-tuned to detect hallucinations in RAG, which allows language models to incorporate external knowledge retrieval mechanisms to enhance their capabilities.

## CONCLUSIONS

Foundation models of future artificial intelligence are able to scale up as the training data get more sophisticated. Modifying these models further can result in outstanding performance across a variety of application domains. Furthermore, corporations have achieved remarkable progress by homogenizing models with LLMs within NLP. Because of their ability to comprehend and manipulate human language, these models are revolutionary and transformative. High-throughput data are integral to these bioinformatics problems: the DNA sequence in genomics, the RNA sequence in transcriptomics, the protein sequence and structure in proteomics, molecular SMILES in drug discovery and multi-omics data in single-cell analysis. Deep learning mechanisms are integrated to acquire biological insights, such as CNN for protein 3D structure features, RNN for time-series single-cell RNA sequencing features, Transformer for biological sequence features and GNN for molecular topology features. By leveraging massive biological datasets, large-scale models are pre-trained and can be applied to a variety of tasks (few shot, zero shot or fine-tuned). Foundation models solve several core biological problems and various downstream tasks rapidly and efficiently.
